# Prioritizing target-disease associations with novel safety and efficacy scoring methods

**DOI:** 10.1038/s41598-019-46293-7

**Published:** 2019-07-08

**Authors:** Mario Failli, Jussi Paananen, Vittorio Fortino

**Affiliations:** Institute of Biomedicine, University of Eastern Finland, Kuopio, Finland

**Keywords:** Target identification, Alzheimer's disease, Drug safety, Data mining

## Abstract

Biological target (commonly genes or proteins) identification is still largely a manual process, where experts manually try to collect and combine information from hundreds of data sources, ranging from scientific publications to omics databases. Targeting the wrong gene or protein will lead to failure of the drug development process, as well as incur delays and costs. To improve this process, different software platforms are being developed. These platforms rely strongly on efficacy estimates based on target-disease association scores created by computational methods for drug target prioritization. Here novel computational methods are presented to more accurately evaluate the efficacy and safety of potential drug targets. The proposed efficacy scores utilize existing gene expression data and tissue/disease specific networks to improve the inference of target-disease associations. Conversely, safety scores enable the identification of genes that are essential, potentially susceptible to adverse effects or carcinogenic. Benchmark results demonstrate that our transcriptome-based methods for drug target prioritization can increase the true positive rate of target-disease associations. Additionally, the proposed safety evaluation system enables accurate predictions of targets of withdrawn drugs and targets of drug trials prematurely discontinued.

## Introduction

The development of a new drug, from target (herein a gene or a protein) identification to Food and Drug Administration (FDA) approval, takes over 10 years and far exceed the cost of $2.6 billion^1^. Most of the costs are sunk into failures, which often occur in late-stage clinical development with a considerable waste of time and money. Reasons for drug failures are mainly safety findings or lack of efficacy in the disease they were intended for. To stem this phenomenon, academic and industrial research has focused on the identification of the most biologically plausible molecular targets that are relevant to the specific disease. This step, often indicated as drug target discovery, represents the first and crucial stage of drug development^2^. Over the last decades, several bioinformatics tools have been implemented to rapidly identify and prioritize genes that encode promising drug targets from public scientific literature and biomedical data^3,4^. In particular, computational methods using large-scale omics data have been proposed. For instance, genome-wide association studies (GWAS) and transcriptome analysis have been successfully used to find target genes for diseases^5–8^, leading to a few cases of new FDA-approved drugs^9,10^. Following the accretion of publicly available data, computational platforms linking potential drug targets to diseases have risen. Some of them, such as Guildify^11^, the Comparative Toxicogenomics Database^12^ (CTD) and DISEASES^13^ focus on evidence based on single data types (e.g. interactome- or literature-based). Others, such as DisGeNET^14^, PHAROS^15^ and Open Targets^16^ (OT) platforms systematically integrate and harmonize multiple biomedical data sources belonging to several data types in order to associate and prioritize potential targets with diseases. In particular, the OT platform detects, scores and integrates evidence-based associations between diseases and putative targets by using a breadth of data types (i.e. genetic associations, somatic mutations, know drugs, gene expression, affected pathways, literature mining and animal models). An important data type is RNA expression, which provides efficacy estimates based on the combination of expression fold change, statistical significance (P-value) and percentile rank. The success of these platforms, in delivering accurate targets, strongly relies on the use of specialized data mining methods enabling the identification of key patterns from large-scale data to be then used for efficacy assessment. However, it is acknowledged that certain methods are more accurate than others^17,18^. For instance, *Ferrero et al*.^17^ have used a machine learning approach to demonstrate that scoring methods derived from different data types used by OT exhibit different prediction performances on known gene disease associations. Besides, efficacy is not the only relevant aspect to be considered. There are in fact other properties that should be examined in the pre-assessment of potential therapeutic targets^19^, such as safety, which is often overlooked by current target discovery platforms^20^.

**Figure 1:**
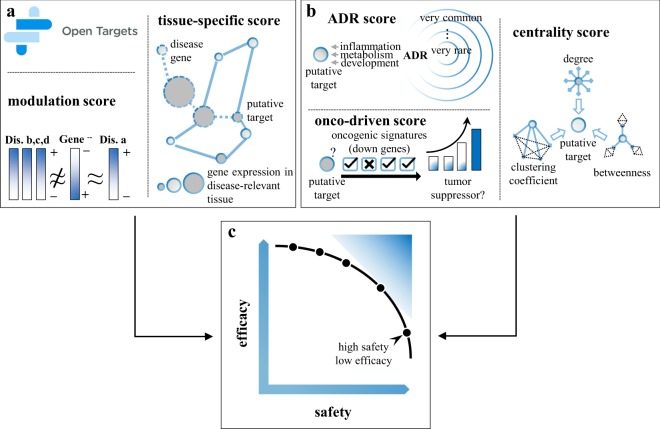
An overview of the proposed methods for drug target prioritization. (**a)** Graphical view of the modulation and tissue-specific scores. **(b)** Graphical view of the ADR, centrality and onco-driven scores. **(c)** Example of trade-offs between efficacy and safety scores.

In this study novel data mining methods are proposed to improve the evaluation of efficacy scores from existing transcriptome data and to complement this information with target safety assessment. Based on the hypothesis that a perturbed gene could result in the correction of transcriptional signatures dysregulated in a particular disease^21^, a new efficacy score, namely *modulation score*, is defined to estimate the likelihood of a gene perturbation (e.g., knockout and knockdown) to result in specific reversion of disease gene-expression profiles. Then, a *tissue-specific efficacy score* is provided under the hypothesis that disease-associated genes are more likely to exhibit tissue-specific expression than non-disease-associated genes^22^. By traversing existing tissue/disease specific networks, the tissue-specific scoring method detects gene targets that are closely related to disease genes in disease-relevant tissues. Moreover, three novel safety scores are introduced to estimate the likelihood of a potential target to be carcinogenic, to develop either common or rare adverse reactions, and to play important roles in biological processes. Figure 1 depicts the general schema of the proposed scoring methods for drug target prioritization. Starting from different sets of known target-disease associations, here referred as to gold standards, the performances of the novel efficacy scores were evaluated alone and in combination with those obtained from OT. Additionally, different benchmarks were compiled in order to accurately test the safety assessment scores. In particular, targets linked to discontinued drugs or drugs failed clinical trials were considered as targets with known safety issues and, therefore, these targets were used to evaluate the prediction capabilities of the propose safety scores. Finally, a case study was conducted to prioritize targets for Type 2 Diabetes (T2D) and Alzheimer’s disease (AD), in order to prove that the proposed scoring methods enable the selection of efficient and safer drug targets.

## Results

### Benchmark datasets for evaluation of drug target prioritization methods

DrugBank^23^ (version 5.1.0) was queried to build a set of known target-disease associations. Medical Subject Headings^24^ (MeSH) terms were systematically matched to the description of DrugBank entries in order to identify known drug-disease associations. These associations were then transformed to target-disease associations by availing of the information on the potential drug targets. Finally, MeSH identifiers were converted to disease EFO^25^ ids (Methods for details). The compiled set of known drug target-disease associations was used to verify whether and to what extend top ranked target-disease associations, resulting from the application of a given efficacy score, matched known associations. In more details, the positive predictive values (ppv) was computed as the percentage of true positives normalized against the expected ppv for a random ordering of target-disease pairs^26^. The ppv curves were supplemented with barplots, which indicate the exact percentage of true positives (TP) obtained with the top n target-disease associations (where n corresponds to the 1^st^, 5^th^ and 10^th^ percentile of the target-disease association list sorted by a given efficacy score). Two additional sets of known target-disease associations were obtained from OT and CTD (see Methods online). Sets of drug targets with potential safety issues were also built in order to evaluate the accuracy of safety assessment scores. A first set of potentially unsafe targets was obtained by selecting targets linked to drugs withdrawn from the market in at least one jurisdiction. Then, targets in clinical trials terminated “prematurely” were obtained from ClinicalTrials.gov by assuming that safety was the main cause of an early termination. Known essential genes in Human^27^ and a derived set of targeted genes in cancer therapies (see Methods) were also considered. In the same way as for the evaluation of efficacy score, ppv curves were measured to evaluate the safety scores. Therefore, drug targets were first ranked based on a given safety scores (from unsafe to safer) and then the top n selected targets were selected and compared with those having known safety issues.

### Efficacy scores derived from Open Target exhibit different accuracy results

**Figure 2:**
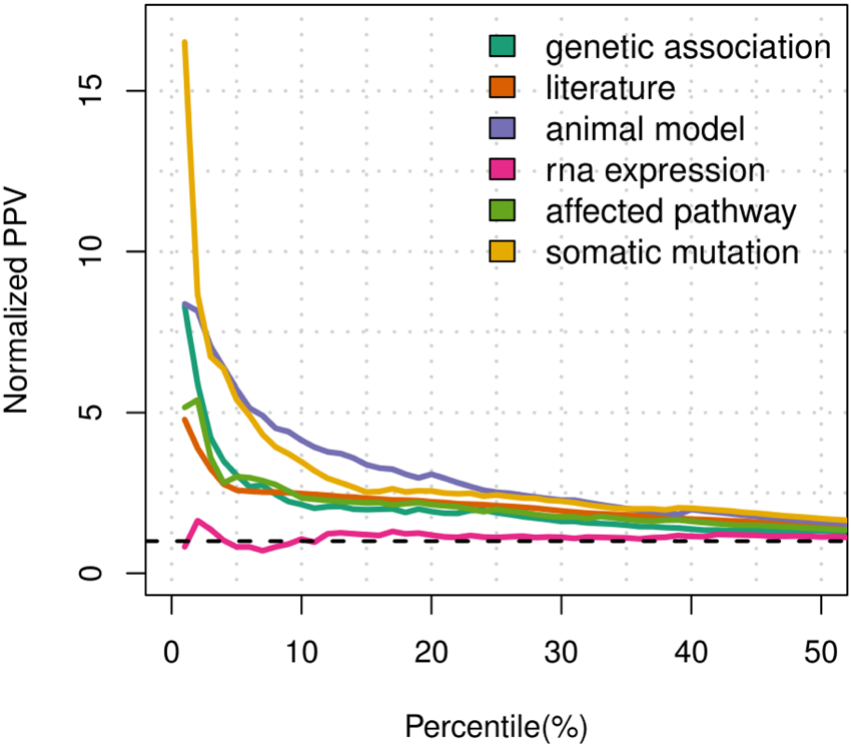
Benchmark results obtained from the individual OT scores. Target-disease pairs having score greater than 0 are sorted based on their efficacy scores for each of the 6 data types available in OT. The sorted lists are then trimmed on the 50^th^ percentile and compared with a set of known target-disease associations in order to obtain the number positive predicted values and calculate the corresponding normalized ppv curves. The dashed line in black indicates the normalized ppv for a random ordering of target-disease pairs.

A set of 1,337,423 direct target-disease associations was retrieved from OT (release 19.02), along with association scores for 20 data sources and 7 data types and an overall score compiled as the sum of the harmonic series of the individual data source scores. These efficacy estimates were then analyzed to understand how the OT platform uses the different target-disease associations to identify and prioritize drug targets. In particular, the number of efficacy estimates covered by the single data types (target-disease association with a score greater than 0), and their combinations (target-disease associations with more than one efficacy estimate), was calculated in order to generate an upset plot indicating the most used data types in OT. The upset plot in Supplementary Fig. S1 shows that almost the 90% of associations are covered by a single data type and that text mining of scientific literature alone covers more than 40% of the entire set of target-diseases associations. Likewise, the number of drug targets (or genes) having at least one OT-score greater than 0 was compiled. This analysis reveals that different data types supply evidence for the same targets (see Supplementary Fig. S2). Figure 2 depicts the ppv curves obtained after benchmarking the OT-driven efficacy scores with known target-disease associations. The positive predictive values show that somatic mutation and genetic association data types performed up to 15.5- and 7.3-fold better than random, respectively. Moreover, RNA-expression performed very poorly (0.6 times better than random at most) suggesting that current scoring methods ranking target-disease associations from transcriptome-data need to be improved. Then, in order to better investigate the poor performance of the RNA-expression scoring method, ppv plots were generated for different therapeutic areas: neurodegenerative, infectious, immune, metabolic, cardiovascular and cancer diseases (see Supplementary Fig. S3). These ppv plots clearly show that the current RNA-based scoring method is not informative for most of the selected therapeutic areas.

### Novel transcriptome-based efficacy scores improve drug target identification

**Figure 3:**
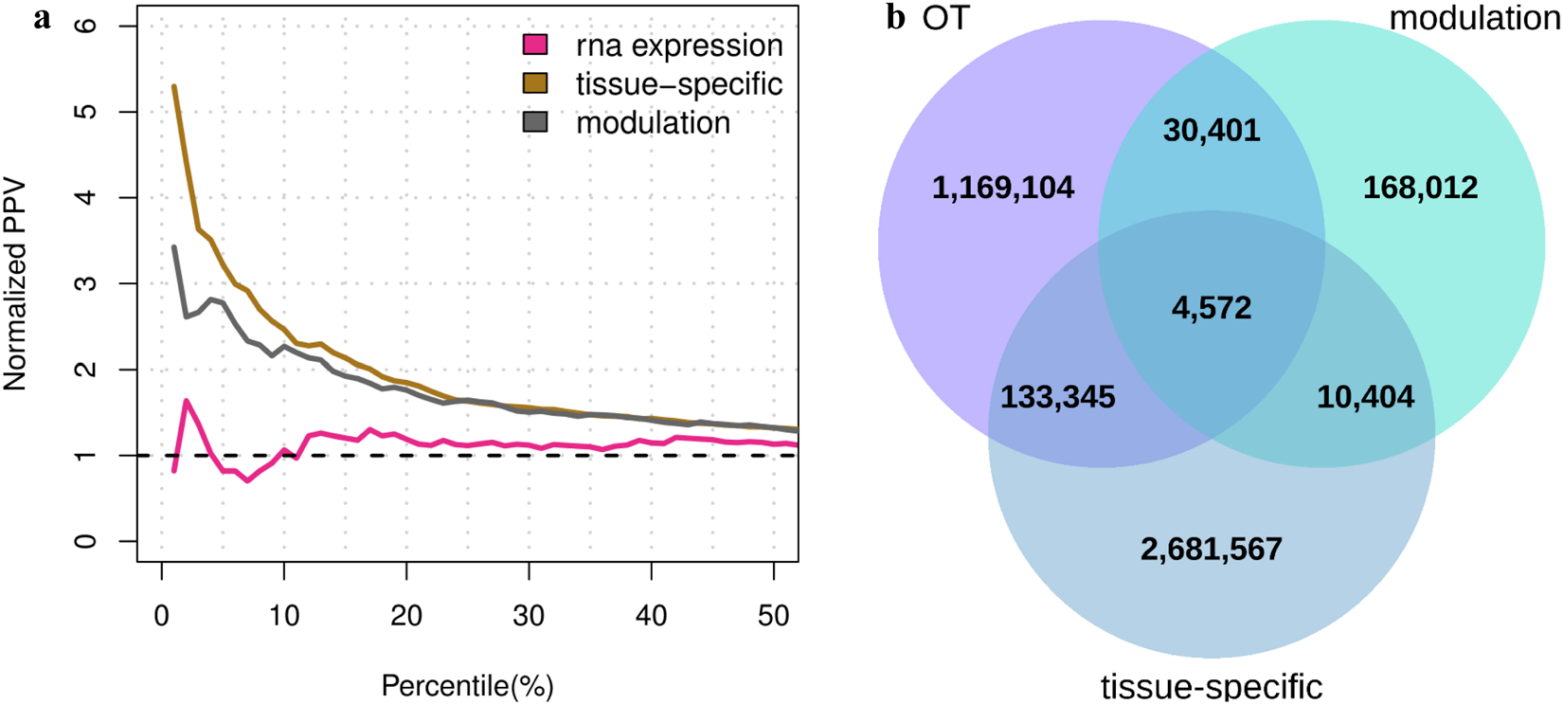
Benchmark results obtained from the novel efficacy scores. (**a**) Ppv curves compiled on the new efficacy estimates for target-disease associations. Target-disease pairs having score greater than 0 are sorted based on the modulation and tissue-specific efficacy scores. The sorted lists are then trimmed on the 50^th^ percentile and compared with a set of known target-disease associations in order to obtain the normalized ppv-curves. The dashed line in black indicates the normalized ppv for a random ordering of target-disease pairs. **(b)** Three-set Venn diagram showing the number of target-disease associations covered by the OT, modulation and tissue-specific efficacy scores.

This study presents two novel computational methods to assess the efficacy of potential drug targets by using existing transcriptome data. A first computational strategy aims to find gene perturbations able to revert the gene expression profile induced by a disease, in order to compile the modulation score, here referred to as *S*_*m*_. First, known lists of up- and down-regulated genes associated with gene perturbations and diseases were downloaded from Enrichr^28^. These lists were then used to compile the number of reversed genes between a given gene modulation and a disease, here referred as to as *C*_*g,d*_. Each count value *C*_*g,d*_, was finally compared with a background distribution in order to calculate its statistical likelihood and to generate a robust efficacy score (see Methods). The second computational strategy instead aims to verify whether a gene is a candidate disease drug target based on its relative distance to known disease genes in tissue-specific gene networks. This score is here referred to as *S*_*t*_. In practical terms, genes that are tied to disease genes through paths including highly tissue-specific genes will tend to have higher efficacy scores (see Methods). The scores proposed for the efficacy evaluation of drug targets were evaluated alone and in combination on a list of known target-disease associations. In particular, ppv curves and barplots, which report the rates of true positives for different percentiles of the score distributions, were used for the comparative analysis. The ppv curves depicted in Fig. 3a show that both the modulation and tissue-specific scores performed better than the RNA-expression data type of OT, by exhibiting 1.8- and 3.7-fold improvements, respectively. Moreover, the proposed efficacy scores lead to new target-disease associations, as shown in Fig. 3b. In addition, ppv curves reporting the rates of two further sets of manually curated and known target-disease associations from CTD and OT, respectively, confirm the improvements of the proposed efficacy scores (see Supplementary Fig. S4). These results, taken together, demonstrate that our efficacy evaluation methods have the ability to detect putative drug targets.

**Figure 4:**
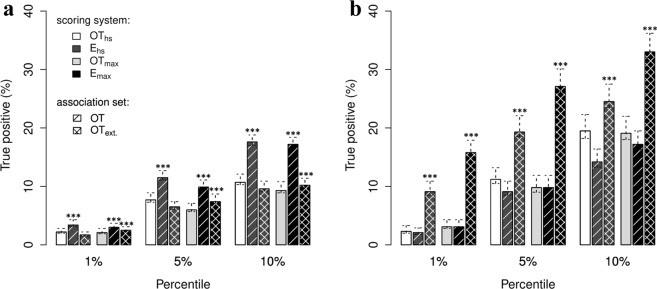
Performance results obtained by combining the proposed efficacy estimates with OT scores. Lists of target-disease associations sorted by the individual efficacy scores were evaluated separately on set of known associations extracted from DrugBank. Rates of TP were determined on both lists *OT* and *OT*_*ext*_. The *OT* contains target-disease associations found by OT; while *OT*_*ext*_ includes novel associations found by the proposed efficacy evaluation systems. These lists were systematically sorted by combining a novel efficacy score with an integration of the OT scores (max or harmonic sum). The sorted lists were then trimmed to the same size in order to have a fair comparison when comparing the rates of TP. **(a)** Barplots indicating the rate of TP obtained from the combination of the modulation score with the OT scores. **(b)** Barplots indicating the rate of TP obtained from the combination of the tissue-specific efficacy score with the OT scores. Each barplot is divided in three sections in order to compare the rates of TP achieved on the 1^st^, 5^th^ and 10^th^ percentile of the sorted target-disease association lists. Each bar indicates the mean TP percentages ± CI are reported for each (n = 100/group; ***fdr < 0.001 *vs*. E_hs_(OT data type) or E_max_(OT data type), one-way ANOVA followed by Tukey’s HSD post-hoc test).

Efficacy scores were also evaluated in combination with those provided by OT. To this end, two different ensemble systems were considered. The first ensemble strategy, here referred to as *E*_*max*_, selects the maximum value between the maximum derived from all OT scores (*OT*_*max*_) and a new efficacy score; *E*_*max*_ = *max(OT*_*max*_, *S*_*m*_) or *E*_*max*_ = *max(OT*_*max*_, *S*_*t*_). The second ensemble strategy, here referred to as *E*_*hs*_, selects the max between the harmonic sum compiled by OT (*OT*_*hs*_) and a new efficacy score; *E*_*hs*_ = *max(OT*_*hs*_, *S*_*m*_) or *E*_*hs*_ = *max(OT*_*hs*_, *S*_*t*_). Note that the overall score obtained from OT was recompiled in order to eliminate the evidence obtained from known drugs. Then, two lists of inferred target-disease associations were selected for the benchmark: one including solely the OT associations and one extending the OT associations with those discovered by the proposed efficacy score (namely *OT*_*ext*_). These lists were subsequently sorted (from highest to lowest), by using the two aforementioned ensemble methods, and trimmed to the same size. Finally, the resulting target-disease association lists were compared against known target-disease associations in order to compile the true positive (TP) rates. Figure 4a,b show the TP rates achieved by using the ensemble methods on the lists *OT* and *OT*_*ext*_. Supplementary Excel File S7 reports the exact differences between each pair of TP rates along with bootstrap confidence intervals. Furthermore, the comparison between the OT system and its extended versions with the modulation and tissue-specific efficacy scores was also carried out with Tukey’s post hoc test to assess if the identified means of true positive rates are significantly different from each other (see Supplementary S7). Barplots in Fig. 4a shows that the ensemble systems including the modulation score perform better than the OT integrated score. In particular, the *E*_*max*_ strategy achieved the best results on the 5^th^ and 10^th^ percentiles of the target-disease associations in OT, increasing the rate of true positives by a factor of 3.882 (549 target-disease associations) and 7.839 (2,217 target-disease associations), respectively. Vice versa, the ensemble systems including the tissue-specific efficacy score obtained the highest TP rates on the extended set of target-diseases associations *OT*_*ext*_, indicating its ability to discover known target-diseases associations that are currently missed by OT. In more details, the *E*_*hs*_ strategy including the tissue-specific score increases the rate of TP by a factor of 6.783 (112 associations) and 8.154 (672 associations) on the 1^th^ and 5^th^ percentiles of the target-disease associations in OT_ext_. Following a similar trend, the *E*_*max*_ strategy increases the rate of TP by a factor of 12.736 (210 associations) and 17.324 (1,427 associations) on OT_ext_. Moreover, no significant increase in performance were found in *E*_*hs*_ and *E*_*max*_ when considering solely OT associations (see Fig. 4b). Supplementary Figs S5–S6 and Supplementary Excel Files S8–S9 report the results for the additional benchmark dataset. To summarize, the benchmark results described in this paragraph demonstrate that the proposed transcriptome-based efficacy scores can be used to improve the accuracy of drug target prioritization platforms and to discover novel target-disease associations.

### Genome-wide data are instrumental in predicting unsafe drug targets

**Figure 5:**
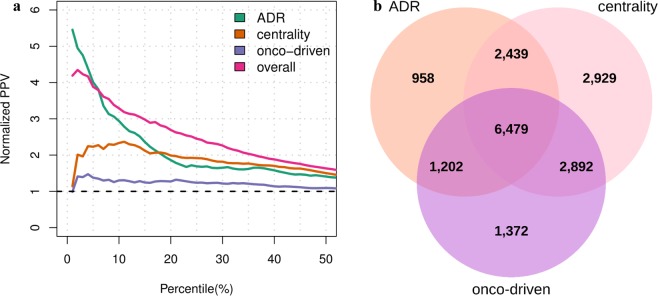
Benchmark results obtained from the safety scores. (**a**) Putative targets are sorted from unsafe to safer according to each safety assessment method. The sorted lists are then trimmed on the 50^th^ percentile and compared with a set of targets with known issues in order to obtain the normalized ppv-curves. The dashed line in black indicates the normalized ppv for a random ordering of target-disease pairs. **(b)** Three-set Venn diagram showing the number of targets covered the different safety scoring systems.

**Figure 6:**
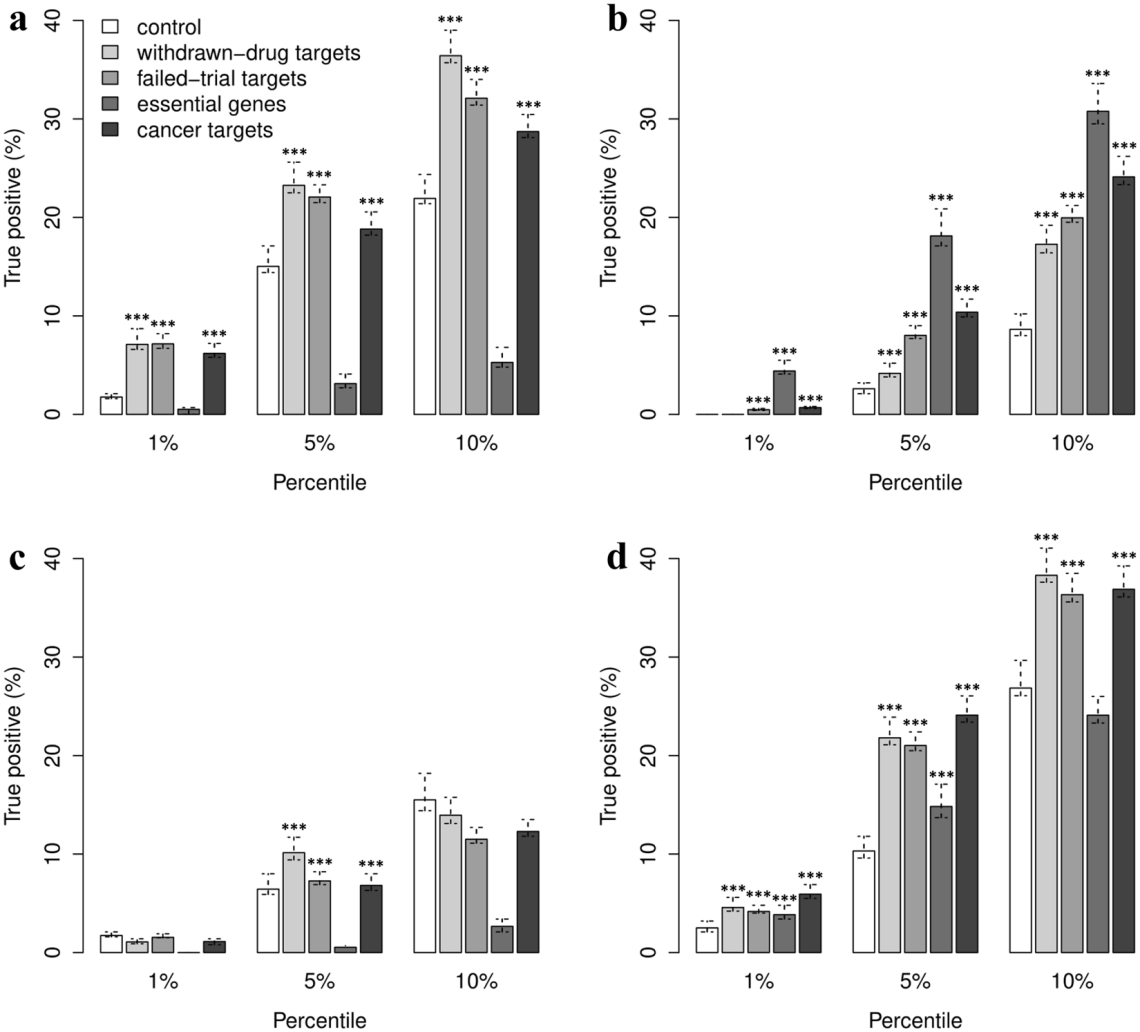
Performance results obtained by testing the safety scores on single benchmarks. Lists of targets sorted by the individual safety scores (from unsafe to safer) were evaluated separately on different categories of potential hazardous targets. These categories include targets of discontinued drugs, targets of drugs failed clinical trials, essential genes and cancer targets. (**a**) Benchmark results obtained by ordering putative targets according to ADR, (**b**) centrality, **(c**) onco-driven and (**d**) overall safety scores. Each barplot reports the TP rates along with bootstrap confidence intervals compiled on the 1^st^, 5^th^ and 10^th^ percentile of the sorted target list (n = 100/group; ***fdr < 0.001 *vs*. control, one-way ANOVA followed by Tukey’s HSD post-hoc test).

A second important aim of this study is to evaluate an OT-like system for the identification and prioritization of potentially safe drug targets. To this end, three safety scores were implemented to assess adverse effects of a drug target (*ADR score*), which were compiled from the ADReCS-Target database^29^, target centrality in tissue-specific gene network (*centrality score*) and whether targets can cause cancer progression when inactivated by a drug (*onco-driven safety score*) (see Methods online). These safety assessment scores were systematically evaluated alone and in combination on sets of targets with known safety issues. In particular, targets associated with drugs withdrawn from market or drugs in clinical trials terminated without results were selected to define sets of drug targets with potential toxic effects. Then, non-conditional essential genes and genes targeted by cancer therapies were also selected (see Methods online). In a similar way as for the assessment of efficacy scores, the benchmarking of safety scores was carried out ordering targets from unsafe to safer. Then, the rate of true positives was compiled for the first top n potentially unsafe targets, with n corresponding to the 1^st^, 5^th^ and 10^th^ percentile of the target list. The ppv curves, depicted in Fig. 5a, show that the ADR and centrality scores perform 5 and 2 folds up, respectively, and that the harmonic sum combining the three safety scores perform 3 folds up for more than the 15^th^ percentile of the target list. Figure 5b shows the number of targets covered by each score and their overlapping sets. These results, taken together, indicate that the proposed safety assessment system for putative drug targets is robust and accurate. Figure 6 depicts a summary of the TP rates achieved by the safety scores against each set of potential unsafe targets. ADR score was highly informative for the prediction of withdrawn-drug targets, failed-trial targets and cancer targets. However, it performed poorly when identifying essential genes (see Fig. 6a). On the other hand, the centrality score reached high rates of TP on the set of essential genes (see Fig. 6b), indicating that an integration of the ADR and centrality score can successfully predict all the defined categories of potentially unsafe targets (see Fig. 6d). There were less significant rates of TP with the onco-driven scores (see Fig. 6c), indicating that this safety score should be improved. A detailed overview of all comparisons between TP rates obtained with different ranking methods for target safety assessment is reported in Supplementary Excel File S10.

### Combining efficacy and safety scores can lead to the discovery of novel drug targets

**Figure 7:**
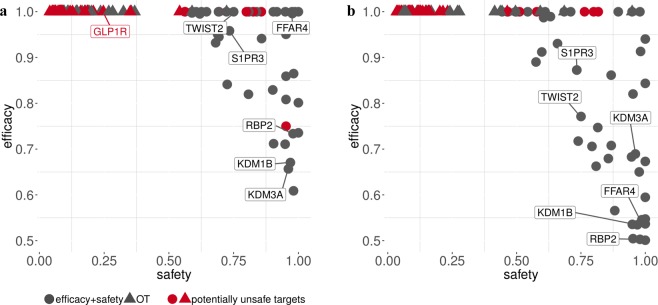
Comparison of the safety and efficacy scores associated to drug targets found for AD and T2D. Scatter plots showing the safety and efficacy scores for the top 50 targets selected by OT and by a weighted scoring system integrating the efficacy (w = 0.5) and safety (w = 0.5) estimates compiled with the new methods. The safety score was derived using a harmonic sum on the ADR, centrality and onco-driven scores. The efficacy score instead was obtained by applying the *E*_*hs*_ ensemble strategy. Targets with known safety concerns are highlighted with the red color. (**a**) Top 50 targets for T2D and (**b**) AD identified by each approach.

A case study aiming to identify and prioritize drug targets for *Type 2 diabetes mellitus* (T2D) and *Alzheimer’s disease* (AD) was conducted in order to show the relevance of combining efficacy with safety scores. According to World Health Organization (WHO) estimates, both T2D and AD are amongst the top ten causes of death worldwide (as at December 2016). Interestingly, pathogenesis and therapeutic similarities have been found between the two diseases^30^. Emerging therapies for the treatment of T2D and AD targeting Glucagon-like peptide-1 receptor (GLP1R) agonists have been proposed^31^. However, a series of adverse effects (acute kidney injury, injection site reactions, headache, and nasopharyngitis) associated with drugs targeting GLP1R and their involvement in cardiovascular events, pancreatitis, or pancreatic cancer^32^, may require the identification of novel drug targets. To this end, the proposed *E*_*hs*_ ensemble system and the complement of the harmonic sum of the three proposed safety scores were calculated to identify putative safer T2D- and AD-targets. The resulting efficacy and safety scores, ranging between 0 and 1, were finally combined with a weighted sum (*W*_*Efficacy*_ = *W*_*Safety*_ = *0.5*) in order to compile an overall score and rank drug targets for T2D and AD. The top 50 selected targets were then compared with those provided by the OT platform. Surprisingly, the targets selected by OT often presented very low safety scores (see Fig. 7). Moreover, the combination of efficacy and safety scores leaded to potential comorbid associations between T2D and AD. In particular, 15 out of 50 putative targets (30%) were shared between the two diseases (Table 1), and three of them (S1PR, GPR120/FFAR4 and RBP2) showed direct evidence of therapeutic involvement in both diseases, based on recent findings and literature. Sphingosine-1-phosphate receptor (S1PR) modulators have been demonstrated to decrease the amyloid-β (Aβ) peptide, the major component of senile plaques deposited in the brains of patients with AD^33^; on the other hand, S1PR blockers have shown to significantly improve glucose tolerance and insulin sensitivity in genetically obese mice, a type 2 diabetes model^34^. Then, G protein-coupled receptor 120 (GPR120/FFAR4) agonists have emerged as promising therapeutic treatments of type 2 diabetes^35^; additionally, the GPR120 agonist docosahexaenoic acid (DHA) has proven to slow the progression of AD^36^. The cellular retinol binding proteins (RBP) have been proposed as potential therapeutic targets for T2D due to their role in adipogenesis^37^; indications of RBP2 as a marker of AD were found too^38^. Other targets shared between T2D and AD were also linked in literature. In particular, the twist family bHLH transcription factor 2 (TWIST2) has been found involved in neuroinflammation and glycogen storage regulations^39,40^, whereas histone H3K4 and H3K9 methylation has been found associated with T2DM and AD progression^41,42^. In this context, both TWIST2 and H3K4 and H3K9 demethylase, such as KDM1B and KDM3A, may represent novel targets for the prevention of both diseases. A list of drugs targeting the selected genes is available in Supplementary Excel File S11. It should be noticed that the case study was conducted by compiling the average between the efficacy and safety scores (which is equivalent to have a weight of 0.5 for both scores). However, in case of life-threatening diseases or highly disabling disorders the efficacy score could be weighted more than the safety score, determining a different target prioritization result. It has already occurred, indeed, that successful drug discovery campaigns of the past have focused more on target efficacy when the efficacy overweighted target safety. A good example is the development of Fingolimod, a sphingosine 1-phosphate receptor 1 (S1PR1) agonist, approved in 2010 for the treatment of *Relapsing-remitting multiple sclerosis* (RRMS) and known for causing a decrease in heart rate^43^. In accordance with this evidence, Fingolimod’s target, S1PR1, was found unsafe by the proposed safety score (overall safety score ~ 0.11). By implication, S1PR1 was detected among the top targets for RRMS only by weighting efficacy more than safety (e.g. W_Efficacy_ = 0.8 and W_Safety_ = 0.2; see Supplementary Excel File S12). Moreover, a counterexample was identified in the development of the proprotein convertase subtilisin/kexin type 9 (PCSK9) inhibitors. PCSK9 inhibitors are a class of drugs approved in 2015 for the treatment of both *Familial hypercholesterolemia* (FH) and *Atherosclerosis* (AS), and not associated with any particular safety risks^44^. The favorable safety profile of their target, PCSK9, was confirmed by the proposed scores (overall safety score~0.79). In addition, PCSK9 placed 6th and 21st among the top targets for AS and FH, respectively, in condition of averaged efficacy and safety (Supplementary Excel File S13).

**Table 1:** Putative targets in common between T2DM and AD among the top 50 identified with the proposed framework.

Gene symbol	Extended name
*AMER1*	APC membrane recruitment protein 1
*B4GALNT2*	beta-1,4-N-acetyl-galactosaminyltransferase 2
*CACNA1S*	calcium voltage-gated channel subunit alpha1 S
*CHRNA9*	cholinergic receptor nicotinic alpha 9 subunit
***FFAR4***	free fatty acid receptor 4
*HMX1*	H6 family homeobox 1
*KDM1B*	lysine demethylase 1B
*KDM3A*	lysine demethylase 3A
*NFIA*	nuclear factor I A
*PCTP*	phosphatidylcholine transfer protein
*PRLH*	prolactin releasing hormone
***RBP2***	retinol binding protein 2
***S1PR3***	sphingosine-1-phosphate receptor 3
*TWIST2*	twist family bHLH transcription factor 2
*ZFP42*	ZFP42 zinc finger protein

## Discussion

This study presents five data mining methods to identify efficient and safer drug targets for specific disease conditions. Two novel methods are proposed to identify and rank target-disease associations by using gene expression profiles in gene modulations and human diseases, and tissue-specific gene expression networks. Furthermore, three safety assessment scores are provided for early detection of potential unsafe targets. Efficacy-driven prioritizations were validated against known target-disease associations obtained from DrugBank; instead, safety-driven rankings of putative drug targets were evaluated on different sets of targets with known safety issues. In particular, targets of withdrawn drugs (retrieved from DrugBank), targets of drugs failed clinical trials (retrieved from ClinicalTrials.gov) and targets corresponding to essential genes were used to test the safety evaluation scores. Benchmark results demonstrated that proposed transcriptome-based scoring methods improve the prediction accuracy of current platforms for drug target prioritization. In particular, they provide better predictions than the OT-gene-expression score. Additionally, the applied methods identified many known target-disease associations that are not annotated in OT. Likewise, safety scores have also proved to make a valuable contribution to the detection of potentially unsafe targets. In this regard, it is interesting to observe that ADR and centrality scores are complementary features and their combination is therefore important to ensure early identification of unsafe drug targets. Finally, a case study was conducted to prioritize and select 50 targets for Type 2 Diabetes (T2D) and Alzheimer’s disease (AD) by using efficacy and safety scores in combination. The main finding of this study is a set of 15 novel target genes that are common between AD and T2D. These targets represent the best trade-offs between efficacy and accuracy and, based on recent findings, they could lead to novel T2D/AD therapeutics. Even though current literature provides support for these genes being potential drug targets for T2D and AD, further studies are obviously needed to study the function and validate the efficacy and safety of these targets.

Overall this study presents novel computational methods for drug-target prioritization that can be used to optimize the selection of putative drug targets based on efficacy and safety pre-evaluations. In particular, the proposed safety assessment scores could be added to current platforms for drug target discovery in order to provide safety information along with efficacy estimates based on target-disease association scores. However, this study represents a first step toward more effective drug target prioritization systems and, most importantly, we believe that novel methods, discovering target-disease associations and related safety issues, should be implemented in order to better leverage the growing volume of omics information for target identification.

## Methods

### Modulation score

The proposed modulation score aims to evaluate whether downstream effects of a perturbed gene can result in the correction of the transcriptional signature dysregulated in a particular disease. First, up- and down-regulated gene lists associated to disease and gene modulations (e.g., knockout and knockdown genes) were downloaded from Enrichr^28^. Then, gene sets associated with experiments performed on animal models were converted to human gene sets by considering the human-mouse equivalent gene names or the human-mouse orthologs (genes with no human orthologs were removed). Starting from the selected gene sets, two separated matrices of gene co-occurrences were created by matching the genes upregulated in diseases with those downregulated in gene perturbations and vice versa. Then, a composite z-score was compiled for each disease-gene perturbation interaction: $f({Z}_{i},{Z}_{j})={Z}_{i}+{Z}_{j};$ where *Z*_*i*_ and *Z*_*j*_ are the *z*-scores from *C*_*i*_, the set of all the co-occurrence values for disease *i* (*row i*), and *C*_*j*_, the set of all the co-occurrence values for gene perturbation *j* (column *j*). Finally, the modulation score between disease *i* and gene perturbation *j* was compiled as:

$$MS(i,j)=\frac{1}{2}(f({Z}_{i},{Z}_{j})^{\prime} +f({Z}_{i},{Z}_{j})^{\prime\prime} )$$

with $f({Z}_{i},{Z}_{j})^{\prime}$ and $f({Z}_{i},{Z}_{j})^{\prime\prime}$ representing the composite z-scores calculated over the two gene co-occurrences matrices. Finally, for each gene-disease pair, only the perturbation giving the maximum score was selected.

$$MS(i,g)=\,{\rm{\max }}\{MS(i,{a}_{1})\ldots MS(i,{a}_{n})\}\forall a\in {P}_{g}$$

with *P*_*g*_ the set of perturbations found in Enrichr for the gene *g*.

### The tissue-specific efficacy score

The second transcriptome-based efficacy score is based on the tissue specificity of human disease genes. In particular, it uses tissue-disease-gene networks to detect shortest paths connecting targets to disease genes by using intermediate genes that are highly tissue-specific. First, genetic association scores provided by OT were used to select the most relevant disease genes (genetic association score equal to 1), here indicated with D. Then, the tissue-specific human interactome published by *Kitsak et al*.^45^ was used to evaluate the paths connecting putative gene targets to disease genes. This interactome consists of 141,296 experimentally supported physical interactions between 13,460 proteins, and gene-expression data for 13,068 genes from 64 non-diseased tissues^45^. For each tissue *t*, a tissue-specific sub-network, here indicated as *I*_*t*_, was formed by selecting the genes that are particularly active in *t* (significance level *z*_*E*_ ≥ *1.0*)^45^. Then, starting from a tissue *t* relevant for the disease *d* the tissue-specificity score of a gene was compiled with the following formula.

$$TSS(D,{I}_{t},g)=-\frac{1}{|D|}\sum _{d\in D}c(d)\ast Dijkstra({I}_{t},g,d)$$

Dijkstra’s algorithm determines the weighted shortest path connecting a putative target *g* to a gene disease. However, in order to apply this algorithm, the node-weighted graph *I*_*t*_ was transformed to an edge-weighted directed graph. In more detail, an edge connecting a *node A* to *node B* was weighted according to the expression level of *B* in the tissue *t* if B was not a disease gene, otherwise the expression level of the node A was used as edge weight. Moreover, since Dijkstra’s algorithm does not work with negative values, the expression values were converted in the form of z-scores scaled in the range [0,1]. Finally, a weighted average was computed on the shortest path scores derived from the best paths connecting a target *g* to each $d\in D$. The weights were compiled in order to reflect the centrality of the destination nodes (or the disease genes). In more details, the Borda aggregation method^46^ was used to rank the disease genes based on three node centrality scores: degree, weighted betweenness and weighted clustering coefficient. Then, a quantile-based discretization method was used to group the genes into equal-sized buckets based on rank and to replace the rank values with discrete levels of node centrality: 0.25, 0.5, 0.75.

### Drug target prioritization based on safety scores

Three scoring methods were proposed to pre-evaluate the safety of gene targets. The first score is based on the hypothesis that genes highly downregulated in cancer can hinder cancer progression, because they might play a causal role in the development of the disease. Therefore, a collection of manually curated gene sets associated with oncogenic pathway activation was download from the Molecular Signatures Database^47^ (MSigDB) oncogenic library. From this library, the gene sets that were highly downregulated under carcinogenic conditions were selected in order to compile the following frequency score for each putative target *g*:

$$onco.driven.score(g)=\,\mathop{\sum }\limits_{s=1}^{S}\,f(g,s),$$

where *f* is the function that returns 1, if a putative target *g* occurs in the set *s*, and 0 otherwise.

The second safety score is based on the idea that genes with high centrality in biological networks have a significantly higher chance of disrupting relevant biological pathways. Therefore, starting from the tissues where diseases were most likely to occur, different centrality scores (degree, weighted betweenness and clustering coefficient) were compiled for each gene within a given tissue-specific interactome *I*_*t*_. The centrality scores were then used to rank the genes, from most (low safety) to least connected (high safety), and the resulting rankings were aggregated with Borda: $R({I}_{t})=Borda({R}_{d}({I}_{t}),{R}_{c}({I}_{t}),{R}_{b}({I}_{t}))$. The ranking outcome obtained from each tissue-specific interactome was finally used to calculate an overall centrality score:

$$centrality.score(g)=\mathop{\sum }\limits_{t=1}^{T}rank\_position(R({I}_{t}),g)$$

The third safety score was implemented under the hypothesis that rare adverse reactions resulting from drug target interactions may have more severe clinical implications than frequent ones. The Adverse Drug Reaction Classification System-Target^29^ database was used to collect adverse drug reactions associated to genes. Then, the ADRs were grouped into 17 high-level categories (HLCs) by etiology (e.g. infections and inflammations), manifestation site (e.g. cardiovascular) or purpose (e.g. emotional). The Supplementary Excel File S14 reports the complete list of the defined categories of ADRs. Therefore, starting from the set of HLC terms associated to a gene *H(g)*, the following formula was applied to determine the probability that a gene can lead to rare adverse reactions as:

$$ADRS(g)=-\,\,\,\,\,\prod _{\forall a\in H(g)}freq(a)$$

Efficacy and safety scores were finally scaled to [0,1], with 1 indicating the most effective targets or the most unsafe targets.

### Selection of known target-disease associations

In order to assess the accuracy of the proposed efficacy scores, a set of 11,439 known target-disease associations was generated by text-matching Medical Subject Headings^24^ (MeSH) terms (or their synonyms), with the description in DrugBank^23^ entries. Disease-drug associations thus identified were then transformed in target-disease associations by replacing drugs with their potential targets retrieved from DrugBank and converting gene symbols to Ensembl IDs. Then the Disease Ontology^48^ (DO) and the Experimental Factor Ontology^25^ (EFO) files were used to map MeSH-IDs to DO-IDs and DO-IDs to EFO-IDs, respectively. The mapping between MeSH-IDs and DOI-IDs was needed to maximize the overlap between MeSH-IDs and EFO-IDs. A second set of 29,265 known target-disease associations was obtained from Open Targets by selecting those associations for which evidence of existing drugs that engage the target and treat the disease were provided. Only drugs that are currently in phase III of clinical trials or marketed were considered at this stage (known drugs score ≥ 0.7). Finally, a third set of 183,959 known target-disease associations was derived from the Comparative Toxicogenomics Database^12^ (CTD). CTD supplies manually curated chemical–gene/protein interactions and chemical–disease associations from the published literature. Thus, the last set of known associations was generated by joining the aforementioned tables by chemical IDs (MeSH-IDs). Since CTD provides both direct and indirect evidence of chemical–gene/protein interactions, only direct interactions (i.e. “chemical binds to protein” or “chemical activates protein”) were considered. Once again, gene symbols were converted to Ensembl IDs and MeSH-IDs to EFO-IDs passing through DO-IDs.

### Selection of drug targets/genes with known safety issues

Four sets of known or putative unsafe drug targets were collected in order to evaluate the performance of the proposed safety scores. The first set of drug targets was built under the assumption that drug treatments, used in interventional studies prematurely terminated or withdrawn without results, might be regarded as potentially unsafe targets. Thus, from ClinicalTrials.gov the top 10,000 studies that matched the aforementioned criteria were selected. Matchings involving placebo or combinations of drugs were excluded. Drug treatments were then crossed with DrugBank (version 5.1.0) entries in order to identify a total number of 598 targets. A second set of potentially unsafe targets was obtained from DrugBank by considering drugs withdrawn from the market in at least one jurisdiction (266 targets). The third set of unsafe targets correspond to the set of (non-conditional) human essential genes extracted from the Online GEne Essentiality^27^ (OGEE) database (183 targets). Finally, the first set of known target-disease associations was used to build two further sets: targets of cancer treatments (454) and targets of non-cancer treatment (234). The set of targets of non-cancer treatment was used as control.

### Statistical analysis

Estimated mean percentages and confidence intervals (P(CI_1_) = 0.25 & P(CI_2_) = 0.975) of true positives were compiled by resampling 100 times the 80% of the gold standard sets. Percentage of each sampled set was established for the top n effective target-disease associations (or the top n unsafe targets), with n corresponding to the 1^st^, 5^th^ or 10^th^ percentile of ordered associations (or targets) according to each efficacy (or safety) score. Differences between TP (sample) means, derived from the bootstrap process, were assessed with a one-way analysis of variance (ANOVA) followed by a Tukey’s honestly significant difference (HSD) test. The ppv curves were systematically normalized against ppv obtained from a random ordering of target-disease associations or, in the case of safety scores, from a random ordering of targets.

## Supplementary information

Supplementary Figures

Supplementary Figures

Supplementary Figures

## Data Availability

The source code for the plots and data (including associations, scores and benchmarks) are available as supplementary materials (CodeAndInputFiles.zip).

## References

[1] DiMasi JA, Grabowski HG, Hansen RW (2016). Innovation in the pharmaceutical industry: New estimates of R&D costs. J. Health Econ..

[2] Hughes J, Rees S, Kalindjian S, Philpott K (2011). Principles of early drug discovery. Br. J. Pharmacol..

[3] Rognan D (2012). Fragment-based approaches and computer-aided drug discovery. Top. Curr. Chem..

[4] Dai Y-F, Zhao X-M (2015). A survey on the computational approaches to identify drug targets in the postgenomic era. BioMed Res. Int..

[5] Mullane K, Winquist RJ, Williams M (2014). Translational paradigms in pharmacology and drug discovery. Biochem. Pharmacol..

[6] Okada Y (2014). Genetics of rheumatoid arthritis contributes to biology and drug discovery. Nature.

[7] Plenge RM, Scolnick EM, Altshuler D (2013). Validating therapeutic targets through human genetics. Nat. Rev. Drug Discov..

[8] Sanseau P (2012). Use of genome-wide association studies for drug repositioning. Nat. Biotechnol..

[9] Cook D (2014). Lessons learned from the fate of AstraZeneca’s drug pipeline: a five-dimensional framework. Nat. Rev. Drug Discov..

[10] Nelson MR (2015). The support of human genetic evidence for approved drug indications. Nat. Genet..

[11] Guney E, Garcia-Garcia J, Oliva B (2014). GUILDify: a web server for phenotypic characterization of genes through biological data integration and network-based prioritization algorithms. Bioinforma. Oxf. Engl..

[12] Mattingly Carolyn J, Colby Glenn T, Forrest John N, Boyer James L (2003). The Comparative Toxicogenomics Database (CTD). Environ. Health Perspect..

[13] Pletscher-Frankild S, Pallejà A, Tsafou K, Binder JX, Jensen LJ (2015). DISEASES: Text mining and data integration of disease–gene associations. Methods.

[14] Piñero, J. *et al*. DisGeNET: a discovery platform for the dynamical exploration of human diseases and their genes. *Database* **2015**, (2015). 10.1093/database/bav028 PMC4397996 25877637

[15] Nguyen D-T (2017). Pharos: Collating protein information to shed light on the druggable genome. Nucleic Acids Res..

[16] Koscielny G (2017). Open Targets: a platform for therapeutic target identification and validation. Nucleic Acids Res..

[17] Ferrero E, Dunham I, Sanseau P (2017). *In silico* prediction of novel therapeutic targets using gene-disease association data. J. Transl. Med..

[18] Zhou H, Skolnick J (2016). A knowledge-based approach for predicting gene–disease associations. Bioinformatics.

[19] Gashaw I, Ellinghaus P, Sommer A, Asadullah K (2011). What makes a good drug target?. Drug Discov. Today.

[20] Harrison RK (2016). Phase II and phase III failures: 2013–2015. Nat. Rev. Drug Discov..

[21] Musa A (2018). A review of connectivity map and computational approaches in pharmacogenomics. Brief. Bioinform..

[22] Kumar V, Sanseau P, Simola DF, Hurle MR, Agarwal P (2016). Systematic Analysis of Drug Targets Confirms Expression in Disease-Relevant Tissues. Sci. Rep..

[23] Wishart DS (2006). DrugBank: a comprehensive resource for *in silico* drug discovery and exploration. Nucleic Acids Res..

[24] MeSH Fact Sheet. Available at, https://www.nlm.nih.gov/pubs/factsheets/mesh.html (Accessed: 30th October 2018).

[25] Malone J (2010). Modeling sample variables with an Experimental Factor Ontology. Bioinforma. Oxf. Engl..

[26] Napolitano F (2018). gene2drug: a computational tool for pathway-based rational drug repositioning. Bioinformatics.

[27] Chen W-H, Minguez P, Lercher MJ, Bork P (2012). OGEE: an online gene essentiality database. Nucleic Acids Res..

[28] Chen EY (2013). Enrichr: interactive and collaborative HTML5 gene list enrichment analysis tool. BMC Bioinformatics.

[29] Huang L-H (2018). ADReCS-Target: target profiles for aiding drug safety research and application. Nucleic Acids Res..

[30] Chatterjee, S. & Mudher, A. Alzheimer’s Disease and Type 2 Diabetes: A Critical Assessment of the Shared Pathological Traits. *Front. Neurosci*. **12** (2018). 10.3389/fnins.2018.00383 PMC6008657 29950970

[31] Sridhar GR, Lakshmi G, Nagamani G (2015). Emerging links between type 2 diabetes and Alzheimer’s disease. World J. Diabetes.

[32] Filippatos TD, Panagiotopoulou TV, Elisaf MS (2014). Adverse Effects of GLP-1 Receptor Agonists. Rev. Diabet. Stud. RDS.

[33] Takasugi N (2013). FTY720/Fingolimod, a Sphingosine Analogue, Reduces Amyloid-β Production in Neurons. PLOS ONE.

[34] Kitada Y (2016). Blockade of Sphingosine 1-Phosphate Receptor 2 Signaling Attenuates High-Fat Diet-Induced Adipocyte Hypertrophy and Systemic Glucose Intolerance in Mice. Endocrinology.

[35] Abdel-Magid AF (2015). Therapeutic Potential of GPR120 Agonists for the Treatment of Type 2 Diabetes. ACS Med. Chem. Lett..

[36] Kim N (2015). Endogenous Ligand for GPR120, Docosahexaenoic Acid, Exerts Benign Metabolic Effects on the Skeletal Muscles via AMP-activated Protein Kinase Pathway. J. Biol. Chem..

[37] Zizola CF (2010). Cellular Retinol-Binding Protein Type I (CRBP-I) Regulates Adipogenesis. Mol. Cell. Biol..

[38] Goodman AB, Pardee AB (2003). Evidence for defective retinoid transport and function in late onset Alzheimer’s disease. Proc. Natl. Acad. Sci. USA.

[39] Mudry JM, Massart J, Szekeres FLM, Krook A (2015). TWIST1 and TWIST2 regulate glycogen storage and inflammatory genes in skeletal muscle. J. Endocrinol..

[40] Su W (2014). The p53 transcription factor modulates microglia behavior through microRNA dependent regulation of c-Maf. J. Immunol. Baltim. Md 1950.

[41] Tu P (2015). Liver histone H3 methylation and acetylation may associate with type 2 diabetes development. J. Physiol. Biochem..

[42] Sanchez-Mut, J. V. & Gräff, J. Epigenetic Alterations in Alzheimer’s Disease. *Front. Behav. Neurosci*. **9**, (2015). 10.3389/fnbeh.2015.00347 PMC4681781 26734709

[43] Akbulak RÖ (2018). Acute and long-term effects of fingolimod on heart rhythm and heart rate variability in patients with multiple sclerosis. Mult. Scler. Relat. Disord..

[44] Bandyopadhyay, D., Ashish, K., Hajra, A., Qureshi, A. & Ghosh, R. K. Cardiovascular Outcomes of PCSK9 Inhibitors: With Special Emphasis on Its Effect beyond LDL-Cholesterol Lowering. *J. Lipids* **2018**, (2018). 10.1155/2018/3179201 PMC5889852 29770231

[45] Kitsak M (2016). Tissue Specificity of Human Disease Module. Sci. Rep..

[46] Saari DG (1999). Explaining All Three-Alternative Voting Outcomes. J. Econ. Theory.

[47] Subramanian A (2005). Gene set enrichment analysis: A knowledge-based approach for interpreting genome-wide expression profiles. Proc. Natl. Acad. Sci..

[48] Schriml LM (2012). Disease Ontology: a backbone for disease semantic integration. Nucleic Acids Res..

