# Publisher Correction: Prioritizing target-disease associations with novel safety and efficacy scoring methods

**DOI:** 10.1038/s41598-020-58426-4

**Published:** 2020-01-31

**Authors:** Mario Failli, Jussi Paananen, Vittorio Fortino

**Affiliations:** Institute of Biomedicine, University of Eastern Finland, Kuopio, Finland

Correction to: *Scientific Reports* 10.1038/s41598-019-46293-7, published online 08 July 2019

Two supplementary files containing tables S7-S14 and the R scripts were omitted from the original version of this Article. This has been corrected in the HTML version of the Article; the PDF version was correct at time of publication.

