# Supplementary material for: Prioritizing target-disease associations with novel safety and efficacy scoring methods
Source: Sci Rep. 2019 Jul 8;9:9852. doi: 10.1038/s41598-019-46293-7 (PMC6614395; doi:10.1038/s41598-019-46293-7)

Prioritizing target-disease associations with novel safety and efficacy scoring methods (Supplementary Figures)

Mario Failli[a], Jussi Paananen[a], and Vittorio Fortino [a,*]

[a] Institute of Biomedicine, University of Eastern Finland, Finland

[*] *To whom the correspondence should be addressed: vittorio.fortino@uef.fi*

Supplementary Figures

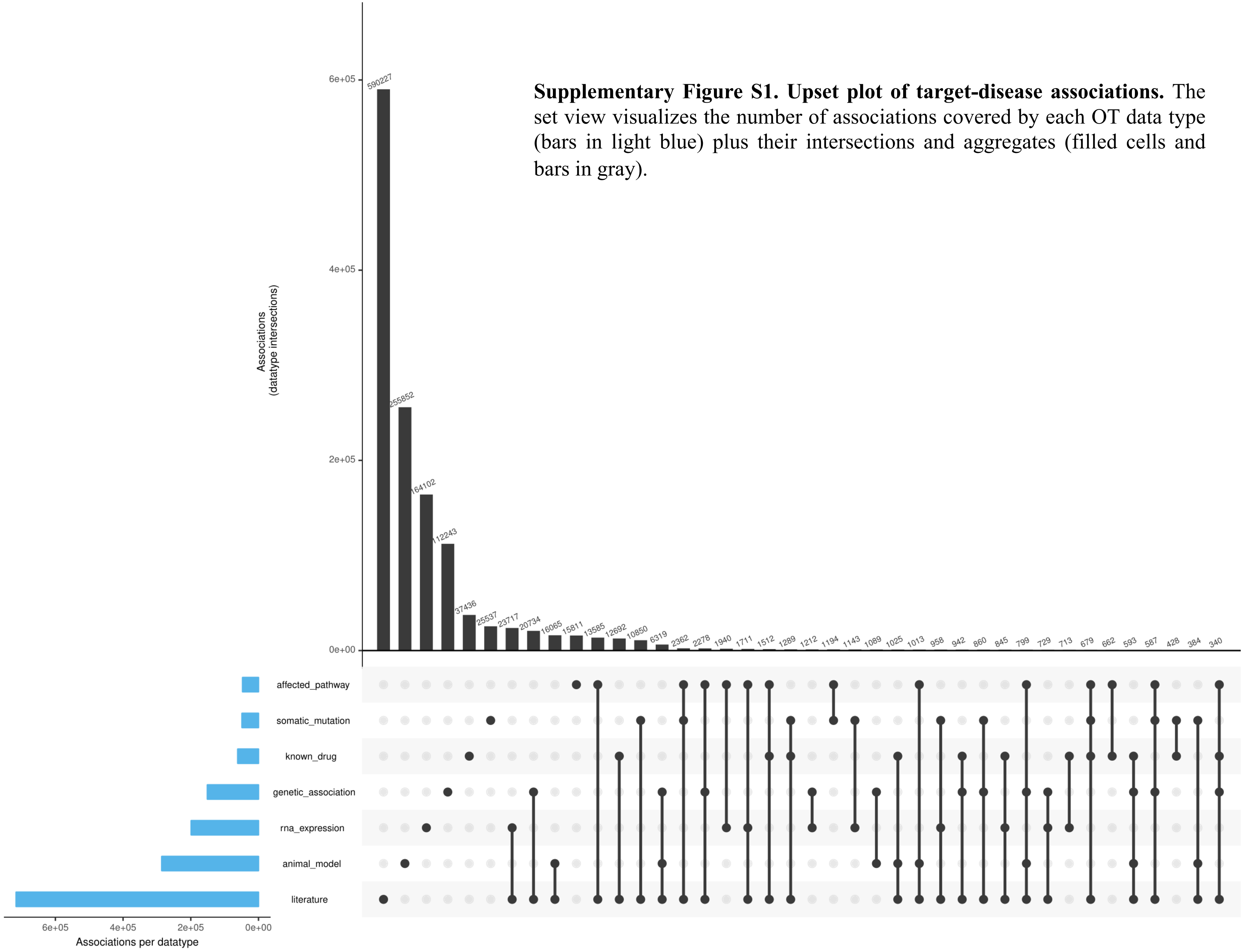

Supplementary Figure S1. Upset plot of target-disease associations. The set view visualizes the number of associations covered by each OT data type (bars in light blue) plus their intersections and aggregates (filled cells and bars in gray).

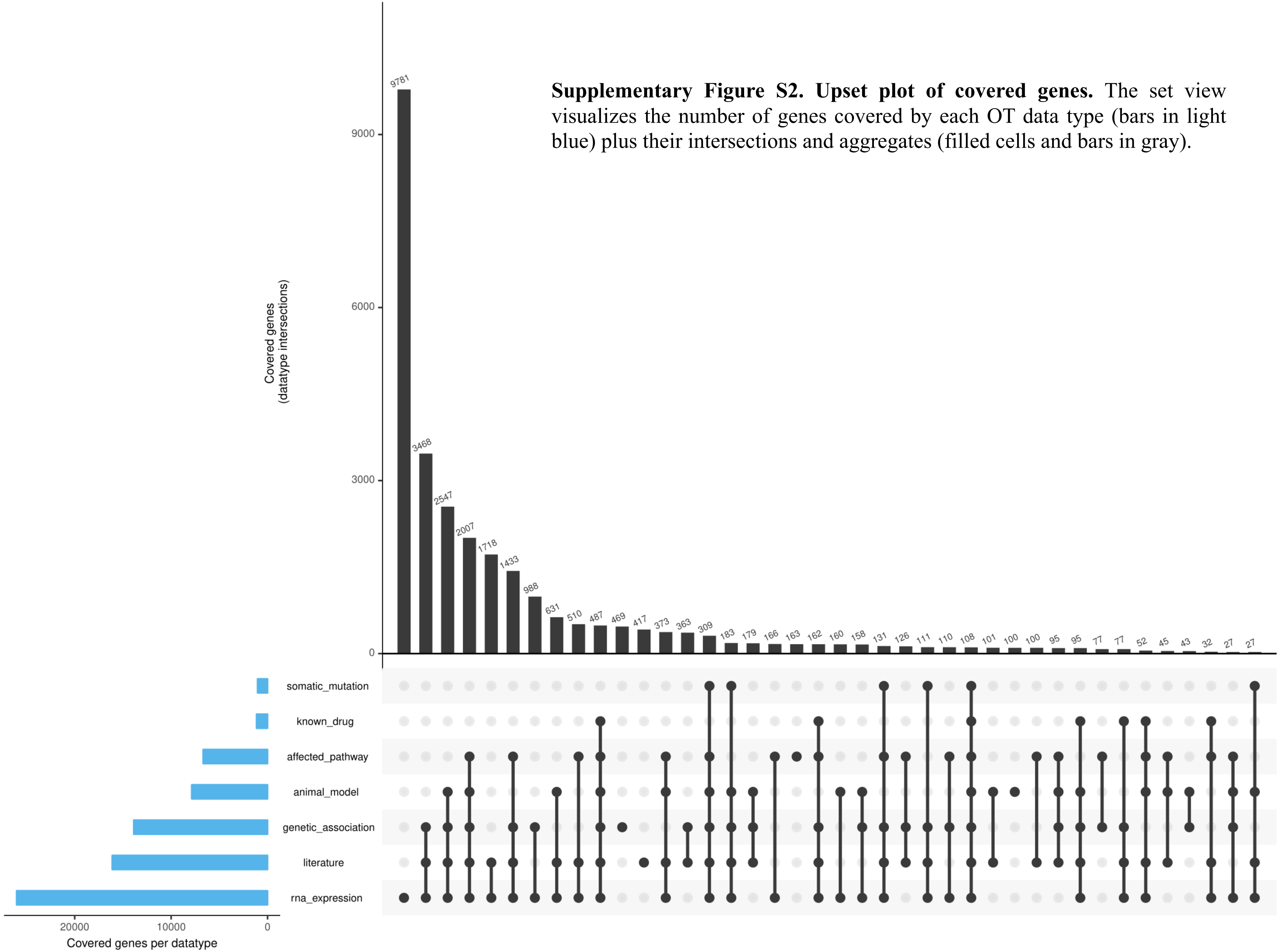

Supplementary Figure S2. Upset plot of covered genes. The set view visualizes the number of genes covered by each OT data type (bars in light blue) plus their intersections and aggregates (filled cells and bars in gray).

Supplementary Figure S3. Benchmark results for six therapeutic areas. Ppv plots for six different therapeutic areas: neurodegenerative(a), infectious(b), immune(c), metabolic(d), cardiovascular(e) and cancer type diseases (f).

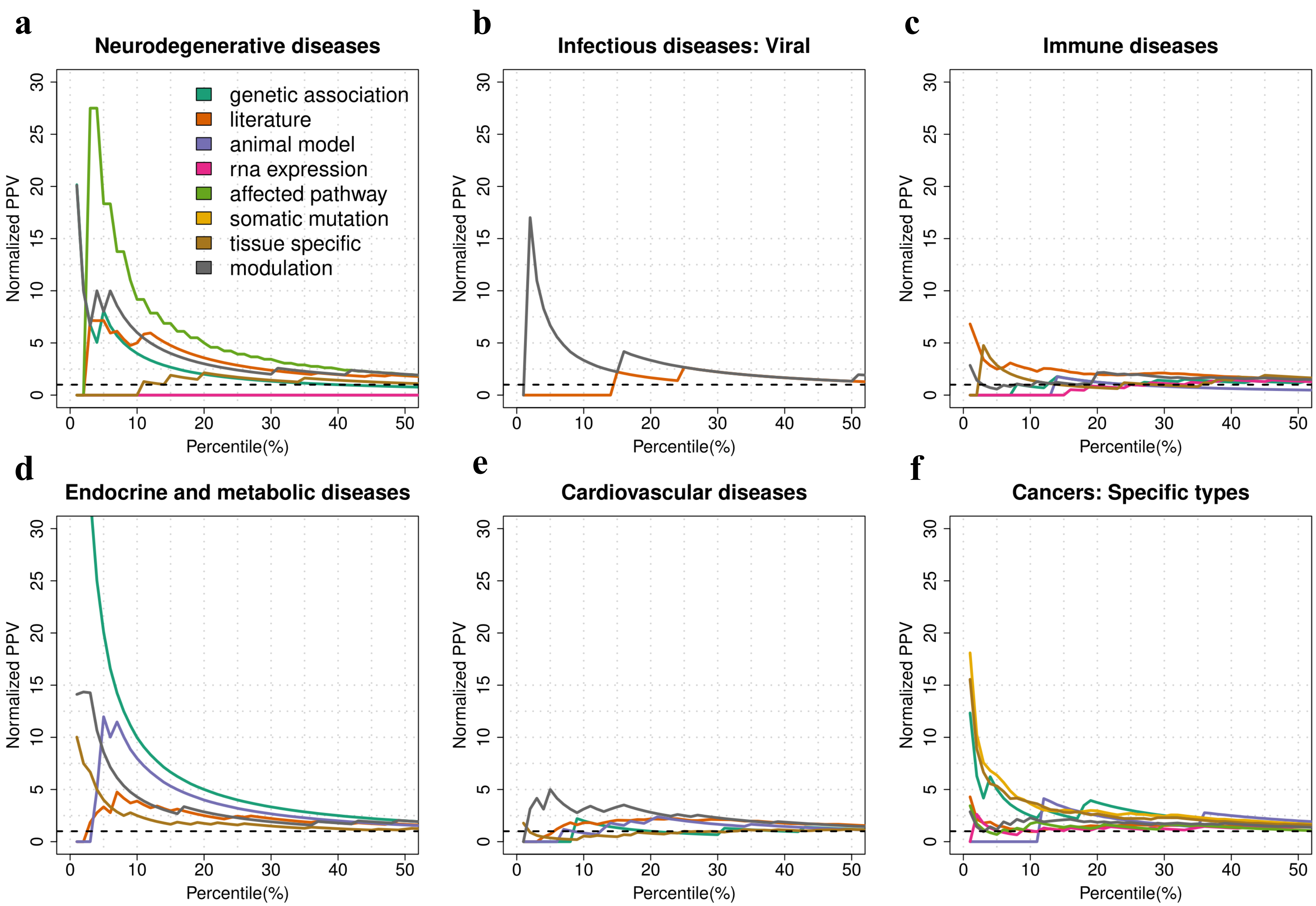

Supplementary Figure S4. Ppv curves compiled on the new efficacy estimates for target-disease associations. Target-disease pairs having score greater than 0 are sorted based on the modulation and tissue-specific efficacy scores. The sorted lists are then trimmed on the 50th percentile and compared with a set of known target-disease associations in order to obtain the normalized ppv-curves. The dashed line in black indicates the normalized ppv for a random ordering of target-disease pairs. **(a)** Comparison with the set of manually curated target-disease associations derived from CTD. **(b)** Comparison with the set of known target-disease associations derived from OT.

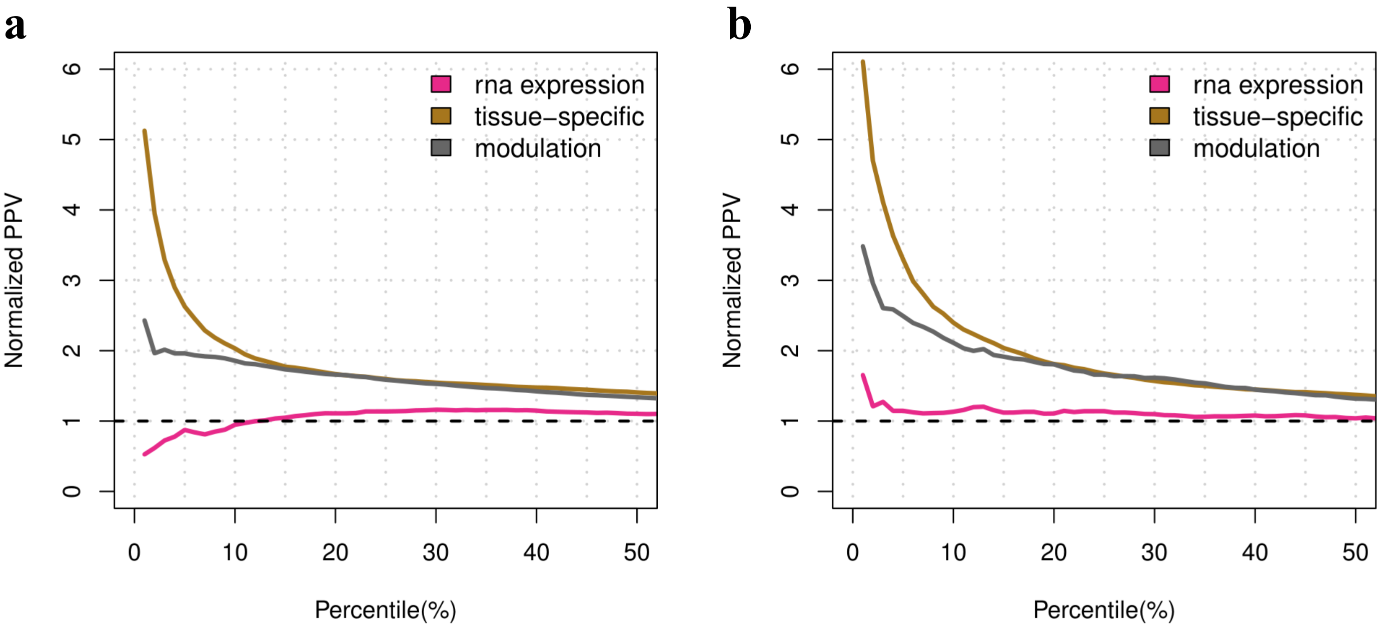

Supplementary Figure S5. Barplots indicating the rate of manually curated target-disease associations derived from CTD. **(a)** Barplots obtained from the combination of the modulation score with the OT scores. **(b)** Barplots obtained from the combination of the tissue-specific efficacy score with the OT scores. Each barplot is divided in three sections in order to compare the rates of TP achieved on the 1st, 5th and 10th percentile of the sorted target-disease association lists. Each bar indicates the mean TP percentages ± CI are reported for each (n = 100/group; ***fdr < 0.001 vs. Ehs(OT data type) or Emax(OT data type), one-way ANOVA followed by Tukey's HSD post-hoc test).

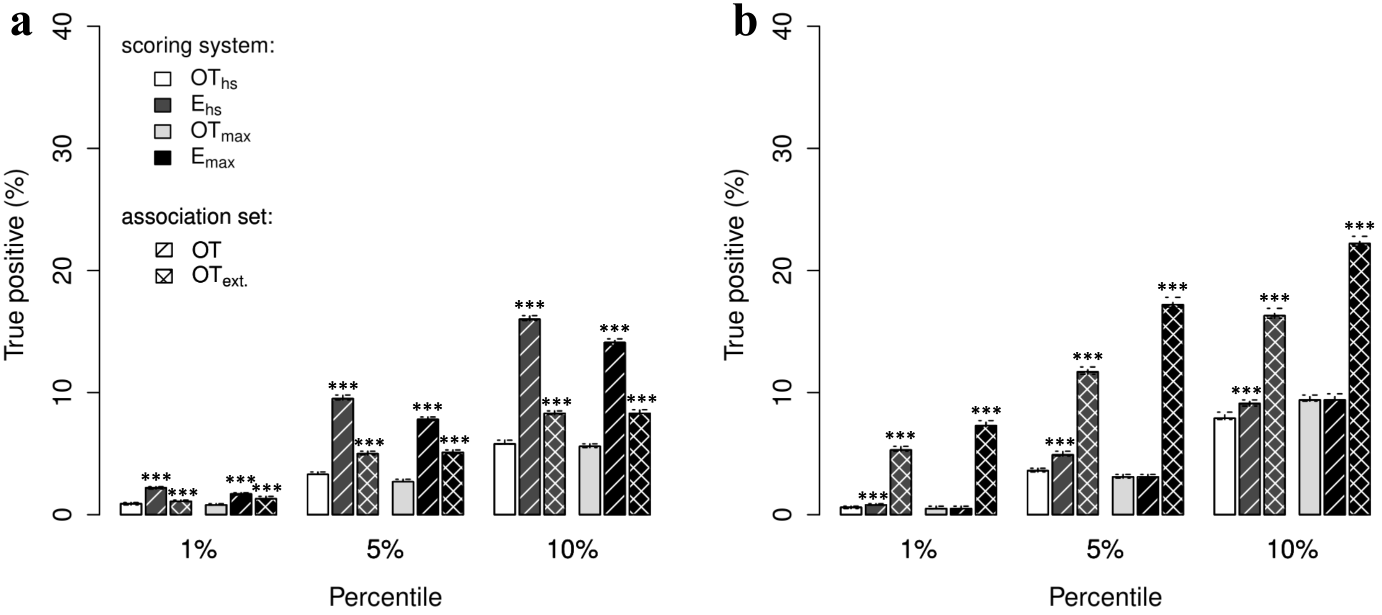

Supplementary Figure S6. Barplots indicating the rate of known target-disease associations derived from OT. **(a)** Barplots obtained from the combination of the modulation score with the OT scores. **(b)** Barplots obtained from the combination of the tissue-specific efficacy score with the OT scores. Each barplot is divided in three sections in order to compare the rates of TP achieved on the 1st, 5th and 10th percentile of the sorted target-disease association lists. Each bar indicates the mean TP percentages ± CI are reported for each (n = 100/group; *fdr<0.05 or ***fdr < 0.001 vs. Ehs(OT data type) or Emax(OT data type), one-way ANOVA followed by Tukey's HSD post-hoc test).

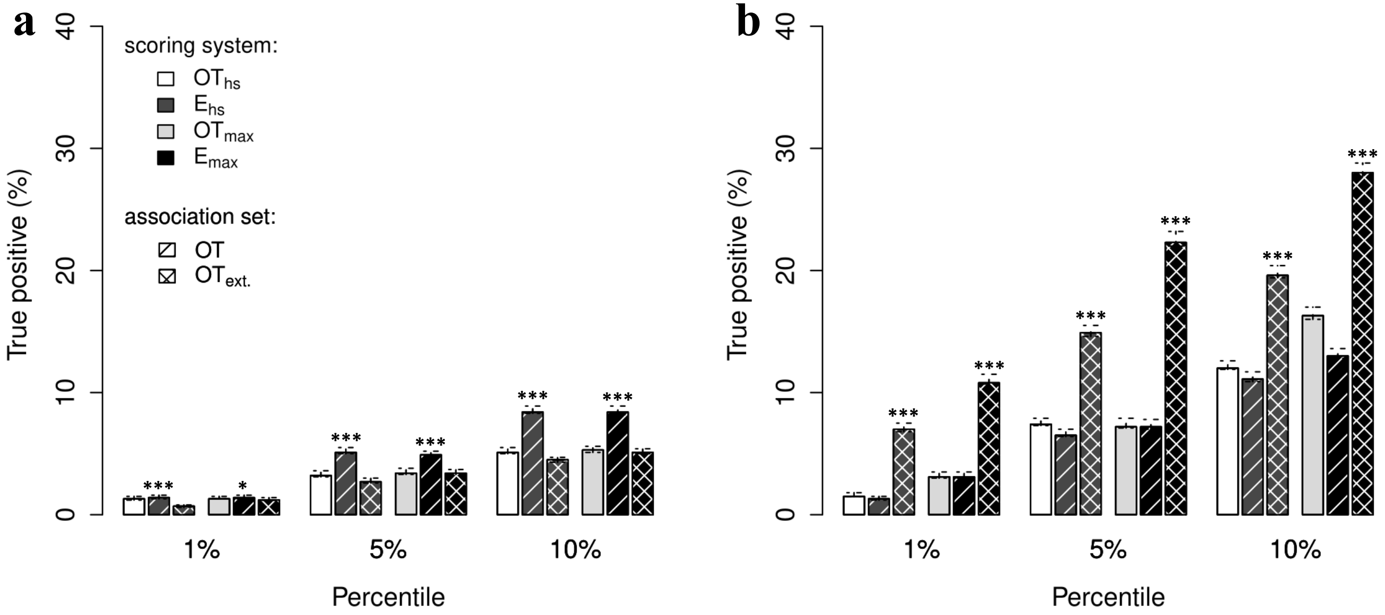

Supplement: Supplementary file 1 — Supplementary Figures [file 41598_2019_46293_MOESM1_ESM.pdf]
