# Supplementary material for: Prioritizing target-disease associations with novel safety and efficacy scoring methods
Source: Sci Rep. 2019 Jul 8;9:9852. doi: 10.1038/s41598-019-46293-7 (PMC6614395; doi:10.1038/s41598-019-46293-7)
Supplement: Supplementary file 2 — Supplementary Figures [file 41598_2019_46293_MOESM2_ESM.zip › Supplementary_Tables.pdf]

Prioritizing target-disease associations with novel safety and efficacy scoring methods (Supplementary Tables)

Mario Failli[a], Jussi Paananen[a], and Vittorio Fortino [a,*]

[a] Institute of Biomedicine, University of Eastern Finland, Finland

[*] *To whom the correspondence should be addressed: vittorio.fortino@uef.fi*

Supplementary Tables (as excel files)

Supplementary Table S7.xlsx

Evaluation results on DrugBank - TP rates and bootstrap confidence intervals of efficacy estimates tested on known target-disease associations derived from DrugBank.

Supplementary Table S8.xlsx

Evaluation results on CTD - TP rates and bootstrap confidence intervals of efficacy estimates tested on known target-disease associations derived from CTD.

Supplementary Table S9.xlsx

Evaluation results on Open Targets - TP rates and bootstrap confidence intervals of efficacy estimates tested on known target-disease associations derived from Open Targets.

Supplementary Table S10.xlsx

Evaluation results for the safety scores - TP rates and bootstrap confidence intervals of safety estimates tested on targets associated with drugs withdrawn from market or drugs in clinical trials terminated without results, non-conditional essential genes and genes targeted by cancer therapies.

Supplementary Table S11.xlsx

List of drugs targeting the genes selected for the case study

Supplementary Table S12.xlsx

Top 50 targets selected for Relapsing-remitting multiple sclerosis (RRMS)

Supplementary Table S13.xlsx

Top 50 targets selected for Familial hypercholesterolemia (FH) and Atherosclerosis (AS)
Supplementary Table S14.xlsx

List of categories for adverse drug reactions - Manually defined categories of ADRs.
