# Supplementary material for: Prioritizing target-disease associations with novel safety and efficacy scoring methods
Source: Sci Rep. 2019 Jul 8;9:9852. doi: 10.1038/s41598-019-46293-7 (PMC6614395; doi:10.1038/s41598-019-46293-7)
Supplement: Supplementary file 3 — Supplementary Figures [file 41598_2019_46293_MOESM3_ESM.zip › Readme.rtf]

Prioritizing target-disease associations with novel safety and efficacy scoring methods. 

Mario Faillia, Jussi Paananena, and Vittorio Fortino a,* 
a Institute of Biomedicine, University of Eastern Finland, Finland
* To whom the correspondence should be addressed: vittorio.fortino@uef.fi
Provided intermidiate files:

1. human_interactome.txt (Table containing the human interactome)

2. gene_expr.txt (Gene expression significance data) 

3. diss_tiss_zscore.txt (File containing all disease tissue pairs with significances)

4. geneXADR.txt (File of gene-ADR associations)

5. MSigDB_Oncogenic_Signatures (Signature of up & down-regulated genes in cancers)

6. Single_Gene_Perturbations.txt (Signature of up & down-regulated genes for perturbed gene) 

7. Disease_Perturbations.txt (Signatures of up & down -regulated genes for disease condition)

8. tissue_score.txt (file containing tissue-specific scores for considered disease-gene associations)

9. modul_score.txt (file containing modulation scores for considered disease-gene associations)

10. opentargets_score.csv (file containing OT data-source and data-type association scores - Release 19.02) 

11. ADR_score.txt (file containing ADR scores for considered genes)

12. centrality_score.txt (file containing centrality scores for considered genes)

13. onco_score.txt (file containing onco-driven scores for considered genes)

14. G.S.txt (file listing known disease-target associations from DrugBank)

15. G.S_open.txt (file listing known disease-target associations from OT)

16. G.S_ctd.txt (file listing known disease-target associations from CTD)

17. withdrawn.txt (file listing targets of withdrawn drugs)

18. clinical_trials_without_results.txt (file listing targets of drugs that have failed in clinical trials)

19. Homo sapiens_consolidated.csv (file reporting the essentialialy status of human genes)

20. is_cancer.txt (file indicating which EFO id corresponds to a cancer disease)

21. targets_associated_with_type_II_diabetes_mellitus.csv (OT overall score for the targets associated with T2DM)

22. targets_associated_with_Alzheimer's_disease.csv (OT overall score for the targets associated with AD)

23. Disease_class.xlsx (Manually curated selection of diseases per therapeutic area)

Provided scripts:

1. efficacy_tissue_specific.R (script used to calculate tissue-specific scores)

2. efficacy_modulation.R (script used to calculate modulation scores)

3. safety_ADR.R (script used to calculate ADR scores)

4. safety_centrality.R (script used to calculate centrality scores)

5. safety_onco.R (script used to calculate onco-driven scores)

6. benchmark_efficacy.R (script used to assess the goodness of proposed efficacy scores)

7. benchmark_safety.R (script used to assess the goodness of proposed safety scores)

8. case_study.R (script used to investigate the case study) 
 
